# The Role of Registers in Increasing Knowledge and Improving Management of Children and Adolescents Affected by Familial Hypercholesterolemia: the LIPIGEN Pediatric Group

**DOI:** 10.3389/fgene.2022.912510

**Published:** 2022-06-20

**Authors:** Marta Gazzotti, Manuela Casula, Stefano Bertolini, Maria Elena Capra, Elena Olmastroni, Alberico Luigi Catapano, Cristina Pederiva, the LIPIGEN Paediatric Group, Massimiliano Allevi

**Affiliations:** ^1^ SISA Foundation, Milan, Italy; ^2^ IRCCS MultiMedica, Sesto San Giovanni (Milan), Italy; ^3^ Department of Pharmacological and Biomolecular Sciences, Epidemiology and Preventive Pharmacology Service (SEFAP), University of Milan, Milan, Italy; ^4^ Department of Internal Medicine, University of Genova, Genova, Italy; ^5^ Centre for Paediatric Dyslipidaemias, Paediatrics and Neonatology Unit, Guglielmo da Saliceto Hospital, Piacenza, Italy; ^6^ Clinical Service for Dyslipidaemias, Study and Prevention of Atherosclerosis in Childhood, Paediatrics Unit, ASST-Santi Paolo e Carlo, Milan, Italy

**Keywords:** familial hypercholesterolemia, pediatric cohort, genetic diagnosis, pathology register, clinical diagnosis, cardiovascular genetics

## Abstract

Pathology registers can be a useful tool to overcome obstacles in the identification and management of familial hypercholesterolemia since childhood. In 2018, the LIPIGEN pediatric group was constituted within the Italian LIPIGEN study to focus on FH subjects under 18 years. This work aimed at discussing its recent progress and early outcomes. Demographic, biochemical, and genetic baseline characteristics were collected, with an in-depth analysis of the genetic defects. The analysis was carried out on 1,602 children and adolescents (mean age at baseline 9.9 ± 4.0 years), and almost the whole cohort underwent the genetic test (93.3%). Overall, the untreated mean value of LDL-C was 220.0 ± 97.2 mg/dl, with an increasing gradient from subjects with a negative (N = 317; mean untreated LDL-C = 159.9 ± 47.7 mg/dl), inconclusive (N = 125; mean untreated LDL-C = 166.4 ± 56.5 mg/dl), or positive (N = 1,053; mean untreated LDL-C = 246.5 ± 102.1 mg/dl) genetic diagnosis of FH. In the latter group, the LDL-C values presented a great variability based on the number and the biological impact of involved causative variants. The LIPIGEN pediatric group represents one of the largest cohorts of children with FH, allowing the deepening of the characterization of their baseline and genetic features, providing the basis for further longitudinal investigations for complete details.

## Introduction

Familial hypercholesterolemia (FH) in its heterozygous form is one of the most common inherited metabolic diseases. It is characterized by elevated serum levels of total cholesterol (TC) and low-density lipoprotein cholesterol (LDL-C) since birth, predisposing to premature atherosclerotic cardiovascular disease (ASCVD) (Fahed and Nemer, 2011; Nordestgaard et al., 2013)

This genetic defect is mainly due to pathogenic variants of the gene encoding for the LDL receptor (*LDLR*), which account for more than 90% of genetically confirmed FH cases and result in reduced uptake and clearance of LDL particles which accumulate in the plasma (Nordestgaard et al., 2013). Less common causes of FH are a few variants of the gene encoding for apolipoprotein B (*APOB*) that interfere with the binding of LDL to its receptor or gain-of-function variants in the gene encoding for proprotein convertase subtilisin/Kexin type 9 (*PCSK9*), a protein involved in the LDLR degradation (Nordestgaard et al., 2013). In the rare autosomal recessive form, variants in the LDL receptor adaptor protein gene (*LDLRAP1*) in the homozygous or compound heterozygous form can also result in an FH phenotype (Fellin et al., 2015; Defesche et al., 2017; Berberich and Hegele, 2019).

A high number of different causative genetic variants with a wide impact on the LDLR expression and function are responsible for the large heterogeneity of LDL cholesterol levels of FH in affected subjects. As such, inheritance of only one mutant allele (heterozygous FH, HeFH) results in a clinical phenotypic expression of variable severity, with physical signs of LDL deposition mainly evident between the third and sixth decade of life (Najam and Ray, 2015), making a prompt diagnosis of FH and treatment more difficult. On the other hand, a genotype with both mutated alleles (homozygous FH and HoFH), either with the same pathogenic variant (true homozygosity) or with different pathogenic variants of the same gene (compound heterozygosity) or of different genes (double heterozygosity), potentially translating into the total absence or defective activity of the LDLR, is characterized by a more severe clinical phenotype and worse prognosis since patients can present with cardiovascular events at a very young age and sometimes also during infancy (Bertolini et al., 2020).

The estimated prevalence of HeFH was reported to be 1:200-500, but recent reports showed a higher prevalence of 1:100-250 albeit not in all populations tested (Akioyamen et al., 2017; Bjornsson et al., 2021). Such a prevalence would expect to yield over 4.5 million patients in Europe and 35 million patients worldwide, of whom 20–25% are children and adolescents. Given the high prevalence of FH, it is estimated that one FH is born every minute (Wiegman et al., 2015; Pyles et al., 2017; Beheshti et al., 2020).

Clinical trials and epidemiology consistently show that lipid-lowering therapies reduce LDL-C and consequently cardiovascular mortality (Mach et al., 2020). Therefore, early detection and treatment in childhood/adolescence are crucial to achieving a normal life expectancy. Familial hypercholesterolemia, however, is still largely underdiagnosed and undertreated (Nordestgaard et al., 2013; Representatives of the Global Familial Hypercholesterolemia Community et al., 2020). Although in adults few diagnostic algorithms are available despite some limitations (Casula et al., 2018), the identification of FH in childhood is made more challenging by the lack of validated diagnostic criteria and the usually less severe physical manifestations of FH in the first decade of life, as a consequence of limited long-life exposure to high concentrations of LDL-C. However, the diagnostic challenges are not the only critical aspect in the management of FH in the pediatric population. Because of their age, children and adolescents need a more dedicated and tailored approach, addressing both the patients and their families, whether it is a dietary approach and lifestyle changes or a pharmacological approach. The need for research to progress both in providing the clinicians with tools to support the diagnosis and in guiding the tailoring of therapeutic interventions is quite compelling (Wiegman et al., 2015).

In the last decade, the implementation of local pathology registers allowed to focus on specific sub-groups as the one including FH subjects under 18 years (Gazzotti et al., 2020; Ramaswami et al., 2020; de Ferranti et al., 2021). National registers of FH play a key role in increasing the detection of patients with FH, understanding gaps in care, and improving management and outcomes. Here, we present the LIPIGEN pediatric group and the early outcomes of its work.

## Methods

In 2018, a subgroup of Italian lipid clinics involved in the LIPIGEN (LIpid TransPort Disorders Italian GEnetic Network) (Averna et al., 2017) study constituted the LIPIGEN pediatric group. The main aim of this sub-study, which includes both pediatric and adult centers that (even occasionally) manage FH subjects under 18 years, was to improve the detection, diagnosis, and management of children and adolescents affected by FH (Averna et al., 2017; Gazzotti et al., 2020).

**FIGURE 1:**
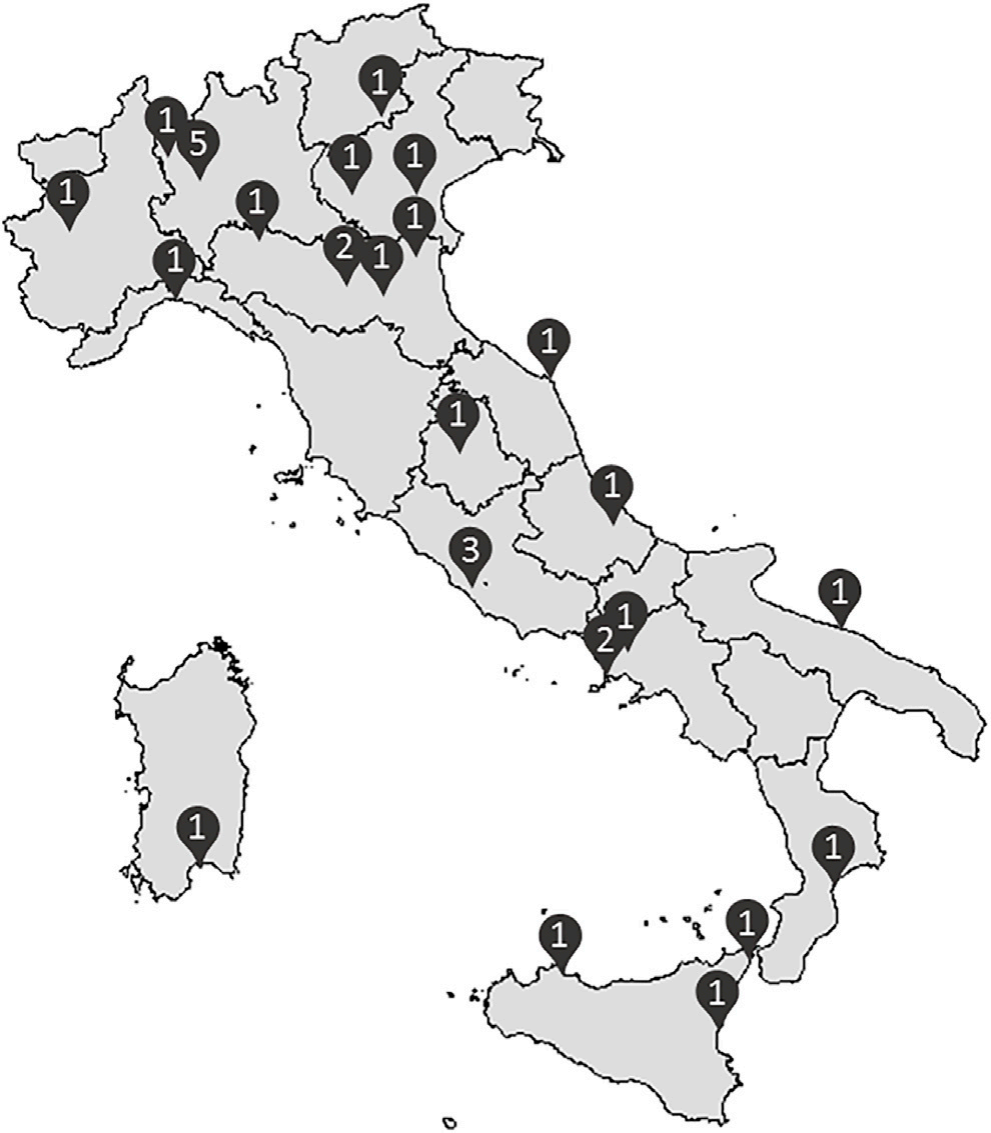
Distribution of the 31 Italian lipid clinics involved in the LIPIGEN pediatric group (number of centers/provinces).

In October 2021, the LIPIGEN pediatric group accounted for more than 1,600 subjects under 18 years with a clinical and/or genetic diagnosis of FH, followed up by 31 LIPIGEN sites in Italy (five of them specifically dedicated to the pediatric population) (Figure 1).

To be enrolled in the LIPIGEN pediatric group, subjects must be under 18 years old at their first presentation at the lipid clinic and receive a clinical and/or genetic diagnosis of FH, according to the specialist’s judgment, in compliance with the LIPIGEN study protocol (Averna et al., 2017). As for the LIPIGEN parental study, demographic, anamnestic, biochemical, and genetic data were collected (Averna et al., 2017). Because of many data being retrospectively collected and referred to historical cohorts, the genetic test was not centralized, and the majority of pediatric samples were analyzed in the local laboratory of each lipid clinic.

Up to 2015, genetic analysis was based on Sanger sequencing of *LDLR, PCSK9,* and *APOB* (apolipoprotein B-binding region of the *APOB* gene to the LDLR). Southern blotting or multiplex ligation-dependent probe amplification (MLPA) was used for the detection of major rearrangements of the *LDLR* gene. Since 2015, the next-generation sequencing (NGS) methodology has been introduced for the simultaneous analysis of the major FH candidate genes (Bertolini et al., 2017; Bertolini et al., 2020); for copy number variations and nucleotide variations in splicing sites, the analysis of transcripts was conducted using Northern blotting and RT-PCR (Bertolini et al., 1999). In the case of compound heterozygous patients with two *LDLR* variants, to differentiate variants in *trans* from those in *cis*, the analysis was extended to the patient’s first-degree relatives to verify familial segregation of the two variants (Bertolini et al., 2020).

We defined a subject as “positive” to the genetic analysis if at least one causative variant, pathogenic or likely pathogenic, was detected according to the ACMG Guidelines (Richards et al., 2015) and the more recent FH-specific suggestions (Chora et al., 2018; Chora et al., 2022); we defined a subject as “inconclusive” if only variants with uncertain clinical significance (VUS) were identified, and “negative” if only benign/likely benign or no variants in the tested genes were found (Richards et al., 2015; Chora et al., 2022) despite the clinical phenotype.

The assignment of pathogenicity was based, in addition to the aforementioned criteria, on *ex vivo* functional characterization of the LDLR activity using either cultured skin fibroblasts or immortalized blood lymphocytes, or *in vitro* LDLR activity validation in cells transfected with LDLR variants (Benito-Vicente et al., 2018), *in silico* analysis of variants using multiple algorithms, and search in the ClinVar database (https://www.ncbi.nlm.nih.gov/clinvar/) and the recently updated *LOVD/LDLR* variant database (https://databases.lovd.nl/shared/genes/LDLR). Furthermore, *LDLR* variants were classified based on their impact on the LDLR activity: values lower than 2–5% were considered to be associated with no receptor activity (null variants), and values higher than 5% were associated with various degrees of reduced receptor activity (defective variants) (Bertolini et al., 1999; Bertolini et al., 2020).

Results for continuous variables are presented as mean (±SD) and median (with IQR), while categorical variables are presented as percentages and numbers. Mean differences between dichotomous variables or among categories were tested using the *t* test or ANOVA, respectively. All analyses were performed using IBM SPSS Statistics 27. Statistical significance was set at the 0.05 level for every analysis performed.

## Results

Overall, the LIPIGEN pediatric register included 1,602 subjects under 18 years with at least one untreated LDL-C measurement and genetic testing data available.

The percentages of males and females were comparable (49.9 and 50.1%, respectively). Two-third of the study population were index cases, while the others were identified through cascade screening, with no difference in the age at baseline (9.8 ± 3.9 vs. 10.0 ± 4.3 years, respectively, *p* = 0.206). Overall, the mean (±SD) age at baseline was 9.9 ± 4.0 years, and the study population was composed of 278 (17.3%) subjects within the age class 0–5 years, 605 (37.8%) in the class of 6–10 years, 381 (23.8%) of 11–13 years, and 338 (21.1%) of 14–17 years (Table 1).

**TABLE 1:** Characteristics of the LIPIGEN pediatric cohort.

Male, N (%)	800 (49.9)
Index cases, N (%)	1,093 (68.2)
Age at baseline [years], mean ± SD	9.9 ± 4.0
Age class 0–5, N (%)	278 (17.3)
Age class 6–10, N (%)	605 (37.8)
Age class 11–13, N (%)	381 (23.8)
Age class 14–17, N (%)	338 (21.1)
On lipid-lowering therapy at the first visit at the LIPIGEN center, N (%)	59 (3.7)

Among the study cohort, 3.7% of subjects were already on lipid-lowering therapy at the first visit to one of the LIPIGEN centers (Table 1). The values of total cholesterol (mean ± SD) were 284.1 ± 98.0 mg/dl among subjects without any lipid-lowering therapy and 255.1 ± 54.2 mg/dl in treated subjects. LDL-C levels (mean ± SD) were 214.0 ± 100.0 mg/dl and 184.4 ± 51.1 mg/dl, HDL-cholesterol levels (mean ± SD) were 53.4 ± 13.6 mg/dl and 54.7 ± 13.5 mg/dl, and triglycerides (median [IQR]) were 75.0 [57.0–100.0] mg/dl and 74.0 [58.0–92.0], respectively (Table 2). Overall, the untreated mean value of LDL-C was 220.0 ± 97.2 mg/dl, with about 90% of individuals in each age group presenting untreated LDL-C >130 mg/dl.

**TABLE 2:** Lipid profile among untreated and treated subjects at the moment of the first visit at the LIPIGEN center.

	Without any lipid-lowering therapy (N = 1,543)	On lipid-lowering therapy (N = 59)
LDL cholesterol [mg/dL], mean ± SD	214.0 ± 100.0	184.4 ± 51.1
Total cholesterol [mg/dL], mean ± SD	284.1 ± 98.0	255.1 ± 54.2
HDL cholesterol [mg/dL], mean ± SD	53.4 ± 13.6	54.7 ± 13.5
Triglycerides [mg/dL], median [IQR]*	75.0 [57.0–100.0]	74.0 [58.0–92.0]

* IQR, interquartile range.

Almost the whole cohort, 93.3% underwent genetic testing to identify the presence of variants in major candidate genes. About 70% of tested individuals (N = 1,053) presented at least one causative variant, while 125 individuals had an inconclusive diagnosis of genetically determined FH (VUS), and for 317 children/adolescents, no known genetic variants were identified to explain the clinical phenotype. Subjects with a positive genetic diagnosis presented higher untreated levels of LDL-C than subjects with only VUS or negative diagnosis (246.5 ± 102.1 mg/dl vs. 166.4 ± 56.5 mg/dl vs. 159.9 ± 47.7 mg/dl, respectively; *p* < 0.0001), while no significant differences in age at baseline were detected.

By stratifying the mean untreated LDL-C levels by genetic diagnosis, values showed variability across age classes despite a significant difference only in the “positive” group (*p* < 0.0001) (Table 3).

**TABLE 3:** Mean ± SD untreated LDL-C levels stratified by genetic diagnosis and age classes.

		Genetic diagnosis		
		Positive	Inconclusive	Negative
Untreated LDL-C [mg/dL]	0–5 years (N = 278)	273.1 ± 128.6	189.2 ± 66.0	173.6 ± 59.0
	6–10 years (N = 605)	248.2 ± 110.4	158.5 ± 40.4	159.1 ± 46.5
	11–13 years (N = 381)	237.3 ± 87.0	169.8 ± 49.4	156.2 ± 43.1
	14–17 years (N = 338)	230.3 ± 66.6	161.1 ± 67.0	157.5 ± 48.7
	Total	246.5 ± 102.1	166.4 ± 56.5	159.9 ± 47.7

Among subjects with a positive diagnosis of FH, 1,015 individuals presented one causative variant in *LDLR* (N = 1,000) or *APOB* (N = 15) genes, while in 38 subjects two causative variants were detected (17 *LDLR* homozygotes, 18 *LDLR* compound heterozygotes, and three double heterozygotes [*LDLR*/*APOB*: N = 2; *LDLR*/*PCSK9*: N = 1]) (Supplementary Tables S1–3). Among subjects with more than one causative variant, the mean untreated LDL-C value was 731.6 ± 154.0 mg/dl in homozygotes, 633.6 ± 211.3 mg/dl in compound heterozygotes, and 229.9 ± 47.0 mg/dl in double heterozygotes, while heterozygotes had a mean LDL-C level of 231.6 ± 53.4 mg/dl (*LDLR* heterozygotes: 231.9 ± 53.3 mg/dl and *APOB* heterozygotes: 208.5 ± 54.9 mg/dl).

In HeFH for *LDLR*, more than 200 different causative variants were detected, with the three most frequently reported being c.1646G > A p. Gly549Asp (N = 93), c.1775G > A p. Gly592GLu (N = 63), and c.662A > G p. Asp221GLy (N = 61). Within each variant, the mean untreated LDL-C levels were 245.0 ± 50.6 mg/dl (min 118 mg/dl, max 400 mg/dl), 198.7 ± 50.1 mg/dl (min 107 mg/dl, max: 367 mg/dl), and 212.2 ± 41.2 mg/dl (min 133 mg/dl, max 302 mg/dl), respectively. The higher levels of LDL-C in the carrier of the former (Supplementary Figure S2, panel A) could be explained by its impact on LDLR residual activity (null variant) compared to the other two receptor-defective variants (Supplementary Figure S1, panels B and C); nevertheless, large variability in the LDL-C values was observed even among carriers of the same variant.

By stratifying *LDLR* causative variants by the receptor residual activity, children/adolescent HeFH with a null variant (<5% residual activity) (N = 470, with more than 120 different variants) presented significantly higher levels of LDL-C than the carriers of a defective-receptor variant (N = 530, with more than 85 different variants): 245.1 ± 52.0 mg/dl vs. 220.2 ± 51.8 mg/dl (*p* < 0.0001), with no differences in the age at baseline (10.0 ± 4.3 vs. 9.8 ± 4.1 years, *p* = 0.432).

## Discussion

Pathology registers have proved to be very useful in providing evidence that can improve knowledge about the pathophysiological basis of diseases and can support clinicians in diagnosis and treatment (Kindt et al., 2017; Gazzotti et al., 2020); this is even more relevant in children. The detection of a genetic severe form of hypercholesterolemia in childhood that implies an accelerated atherosclerotic process can lead to a prompt lipid-lowering treatment in order to reduce coronary heart disease in young adult age (Wiegman et al., 2015).

Recently, various pathology-specific registers for genetic dyslipidemia have been implemented (Collaboration et al., 2018). The LIPIGEN pediatric project is part of a well-established National Network (LIPIGEN project) aiming at improving and enhancing the knowledge of genetic dyslipidemia starting from the first decade of life (Averna et al., 2017). Data from our cohort can fully fit into the pathology register landscape (Kodra et al., 2018; Collaboration, 2021) as the UK National Pediatric Familial Hypercholesterolemia Register (Ramaswami et al., 2017), the Czech MedPed registry (Vrablik et al., 2017), or the Greek Pediatric FH Register (Mollaki et al., 2013). These local experiences and their incorporation into international collaborations of country-specific databases, such as the International Pediatric FH register (Ramaswami et al., 2020), result in a large-scale real-world data collection, necessary to fill the gaps in knowledge, and allow to compare different approaches on identification and treatment of FH.

In our cohort, genetic analysis has been performed on 93.3% of subjects. A pathogenic variant has been identified in 70% of children/adolescents, so the percentage of patients under 18 years with a genetic confirmation is comparable to that highlighted in other European pediatric cohorts, such as the UK (67%), but lower than the Czech cohort (85%) and the pooled pediatric data from eight European countries (88%), where 93.5% of patients had genetic analysis performed (Futema et al., 2021). This variability strongly depends on the inclusion criteria and on the screening strategy implemented in each country. In Italy, the current screening strategy is not universal screening but targeted cascade screening for the identification of potential FH subjects.

The characteristics of the Italian cohort are very similar to those of other pediatric cohorts, for example, in terms of the mean age at diagnosis (10 years old in LIPIGEN, a range of 8–11 years in the European cohorts (Ramaswami et al., 2020), and 9 years in the CASCADE-FH Registry youth participants (de Ferranti et al., 2021)). In fact, in these cohorts, subjects are identified both through opportunistic screening and cascade screening starting from index cases in the family. Instead, much lower is the average age at diagnosis in those cohorts that provide for systematic screening in pre-school children, as in Greece (3 years) (Mollaki et al., 2013) and Slovenia (6 years) (Groselj et al., 2018).

Mean levels of untreated LDL-C were also comparable to those of other pediatric cohorts: 220 mg/dl in LIPIGEN, a range of 188–240 mg/dl in the European cohorts (Ramaswami et al., 2020), and 238 mg/dl in the CASCADE-FH Registry youth participants (de Ferranti et al., 2021)). Moreover, also in our study population, the most common cause of FH was the presence of at least one causative pathogenic variant in the *LDLR* gene while the prevalence of carriers of a heterozygous causative variant on the *APOB* gene was 1.4%. The most prevalent variant in our Italian pediatric cohort was c.1646G > A p. Gly549Asp, a disruptive-missense variant that showed reduced LDL uptake in an *in vitro* study (Bertolini et al., 1999; Thormaehlen et al., 2015); this is one of the most frequent variants also among LIPIGEN adults (untreated mean LDL-C 275 mg/dl). In LIPIGEN children carrying this null variant, the mean untreated LDL-C level was 245 mg/dl, similar to those detected in other European cohorts (256 mg/dl) (Futema et al., 2021). A lower mean untreated LDL-C value was detected among LIPIGEN children/adolescents with the defective variant p. Gly592GLu (198 mg/dl) but still comparable with carriers from another European cohort (199 mg/dl). Moreover, the stratification of *LDLR* heterozygous variants by LDLR residual activity confirmed in children a more severe phenotype among carriers of a null variant compared to carriers of a defective variant (Bourbon et al., 2017).

The large sample size is one of the strengths of our study that enables us to provide an accurate report of the Italian scenario. The presence of the LIPIGEN network as the root of the LIPIGEN pediatric group is an added value as the LIPIGEN network is well-established and geographically well-distributed in all the country, involving lipid clinics with long-time experience. In these centers, expert lipidologists have been dealing with genetic dyslipidemias for decades, so they have been able to properly carry on the data collection, support analysis strategies and also suggest modulation of the collection of family and medical history, adding specific pediatric items to the standard clinical and biochemical data collection (e.g., family history extended to second-degree relatives, the lipid profile of the affected parent, and the age of puberty onset). The combination of these new data with the ones that are being collected about the follow-up of FH pediatric patients will also allow evaluating longitudinal outcomes, such as the effect of lipid-lowering therapies in this age group and the response to treatment, in terms of real-life effectiveness and safety.

The main limitation to our register is that by its nature, it is an opportunistic sample and is likely to be biased toward children from more severely affected families than FH children in the general population. Moreover, the partially retrospective nature of the data collection generated a certain number of missing data. However, researchers are actively working toward information retrieval and standardization.

In conclusion, data collected in the LIPIGEN pediatric network are consistent with those of the main pathology specific registers and allow to better focus on children and adolescents with FH, characterizing their baseline and genetic features, providing the basis for further longitudinal investigations and representing an opportunity to deepen the analysis of the genotype and phenotype of children with FH. The collection of follow-up information and the implementation of the register will help us in improving diagnosis and management standards for FH in Italy starting from childhood.

## LIPIGEN Paediatric Group

Massimiliano Allevi, Internal Medicine and Geriatrics, Department of Clinical and Molecular Sciences, University “Politecnica delle Marche” and IRCCS-INRCA, Ancona, Italy; Marcello Arca, Dipartimento di Medicina Traslazionale e di Precisione, Sapienza Università di Roma—A. U. O Policlinico Umberto I, Rome, Italy; Renata Auricchio, Dipartimento di Scienze Mediche Traslazionali, AOU Policlinico Federico II, Naples, Italy; Maurizio Averna, Dipartimento di Promozione della Salute, Materno-Infantile, di Medicina Interna e Specialistica di Eccellenza, Università degli Studi di Palermo, Palermo, Italy; Davide Baldera, Dipartimento di Scienze Biomediche, Università degli Studi di Cagliari and Centro per le Malattie Dismetaboliche e l’Arteriosclerosi, Associazione ME.DI.CO. Onlus Cagliari, Cagliari, Italy; Giuseppe Banderali, U.O. Clinica Pediatrica, Servizio Clinico Dislipidemie per lo Studio e la Prevenzione dell’Aterosclerosi in età Pediatrica, ASST-Santi Paolo e Carlo, Milan, Italy; Andrea Bartuli, UOC Malattie Rare e Genetica Medica, Ospedale Pediatrico Bambino Gesù, IRCCS, Rome, Italy; Stefano Bertolini, Department of Internal Medicine, University of Genova, Genova, Italy; Giacomo Biasucci, Centro Dislipidemie in Età Evolutiva, U.O. Pediatria e Neonatologia, Ospedale Guglielmo da Saliceto, Piacenza, Italy; Claudio Borghi, U.O. di Medicina Interna Cardiovascolare, Centro Aterosclerosi, Ambulatorio Dislipidemie, IRCCS S. Orsola Ospedale Policlinico S. Orsola-Malpighi, Bologna, Italy; Patrizia Bruzzi, U.O.C. Pediatria, Azienda Ospedaliero Universitaria di Modena, Modena, Italy; Raffaele Buganza, Paediatric Endocrinology, Department of Public Health and Paediatric Sciences, Turin University, Turin, Italy; Paola Sabrina Buonuomo, UOC Malattie Rare e Genetica Medica, Ospedale Pediatrico Bambino Gesù, IRCCS, Rome, Italy; Paolo Calabrò, U.O.C. Cardiologia Clinica a Direzione Universitaria e U.T.I.C., A.O.R.N. “Sant'Anna e San Sebastiano”, Caserta, Italy and Dipartimento di Scienze Mediche Traslazionali, Università degli Studi della Campania "Luigi Vanvitelli”, Naples, Italy; Sebastiano Calandra, Department of Biomedical, Metabolic and Neural Sciences, University of Modena and Reggio Emilia, Modena, Italy; Maria Elena Capra, Centro Dislipidemie in Età Evolutiva, U.O. Pediatria e Neonatologia, Ospedale Guglielmo da Saliceto, Piacenza, Italy; Francesca Carubbi, U.O. Medicina interna metabolica, Centro dislipidemie e malattie metaboliche rare, Ospedale Civile Baggiovara, AOU di Modena, Modena, Italy; Manuela Casula, Dipartimento di Scienze Farmacologiche e Biomolecolari, Università degli Studi di Milano, and IRCCS MultiMedica, Sesto San Giovanni (Milan), Italy; Alberico Luigi Catapano, Dipartimento di Scienze Farmacologiche e Biomolecolari, Università degli Studi di Milano, and IRCCS MultiMedica, Sesto San Giovanni (Milan), Italy; Arturo Cesaro, U.O.C. Cardiologia Clinica a Direzione Universitaria e U.T.I.C., A.O.R.N. “Sant'Anna e San Sebastiano”, Caserta, Italy and Dipartimento di Scienze Mediche Traslazionali, Università degli Studi della Campania “Luigi Vanvitelli”, Naples, Italy; Francesco Cipollone, Clinica Medica, Centro di riferimento regionale per le Dislipidemie, Ospedale Policlinico S.S. Annunziata, Chieti, Italy; Nadia Citroni, Centro Dislipidemie e Aterosclerosi, UOC Medicina Interna, Ospedale di Trento, Trento, Italy; Giuseppe Covetti, U.O. Medicina Interna 2, Centro per le malattie da arteriosclerosi, AORN Cardarelli, Naples, Italy; Annalaura Cremonini, IRCCS Ospedale policlinico San Martino UOSD Dietetica e Nutrizione Clinica and Dipartimento di Medicina Interna, Università di Genova, Genova, Italy; Sergio D’Addato, U.O. di Medicina Interna Cardiovascolare, Centro Aterosclerosi, Ambulatorio Dislipidemie, IRCCS S. Orsola Ospedale Policlinico S. Orsola-Malpighi, Bologna, Italy; Maria Del Ben, Dipartimento Scienze Cliniche, Internistiche, Anestesiologiche e Cardiovascolari - Sapienza Università, A.O. Policlinico Umberto I, Rome, Italy; Maria Donata Di Taranto, Dipartimento di Medicina Molecolare e Biotecnologie Mediche, Università degli studi di Napoli Federico II and CEINGE Biotecnologie Avanzate s.c.a.r.l., Naples, Italy; Giuliana Fortunato, Dipartimento di Medicina Molecolare e Biotecnologie Mediche, Università degli studi di Napoli Federico II and CEINGE Biotecnologie Avanzate s.c.a.r.l., Naples, Italy; Roberto Franceschi, UOC Pediatria, Ospedale di Trento, Trento, Italy; Federica Galimberti, IRCCS MultiMedica, Sesto San Giovanni (Milan), Italy; Marta Gazzotti, Fondazione SISA (Società Italiana per lo Studio dell’Aterosclerosi), Milan, Italy; Simonetta Genovesi, IRCCS Istituto Auxologico Italiano and Dipartimento di Medicina e Chirurgia, Università di Milano-Bicocca, Milan, Italy; Antonina Giammanco, Dipartimento di Promozione della Salute, Materno-Infantile, di Medicina Interna e Specialistica di Eccellenza, Università degli Studi di Palermo, Palermo, Italy; Liliana Grigore, Centro per lo Studio dell'Aterosclerosi, IRCCS MultiMedica, Sesto San Giovanni (Milan), Italy and Centro per lo Studio dell’Aterosclerosi, Ospedale E. Bassini, Cinisello Balsamo, Milan, Italy; Ornella Guardamagna, Paediatric Endocrinology, Department of Public Health and Paediatric Sciences, Turin University, Turin, Italy; Arcangelo Iannuzzi, U.O. Medicina Interna 2, Centro per le malattie da arteriosclerosi, AORN Cardarelli, Naples, Italy; Gabriella Iannuzzo, Dipartimento di Medicina Clinica e Chirurgia, Centro Coordinamento regionale per le Iperlipidemie, AOU Policlinico Federico II, Naples, Italy; Lidia Lascala, AOU Mater Domini, Catanzaro; Fabiana Locatelli, Ambulatorio ipertensione dislipidemie rischio cardiovascolare, ASST Valle Olona, Ospedale di Gallarate, Gallarate, Italy, Ospedale di Busto Arsizio, Busto Arsizio, Italy; Lorenzo Iughetti, U.O.C. Pediatria, Azienda Ospedaliero Universitaria di Modena, Modena, Italy; Sara Madaghiele, U.O. di Medicina Interna e Geriatria “C. Frugoni” and Centro di Assistenza e Ricerca Malattie Rare, A.O. Universitaria Policlinico Consorziale, Università degli Studi di Bari “Aldo Moro”, Bari, Italy; Giuseppe Mandraffino, Department of Clinical and Experimental Medicine—Lipid Center—University Hospital G. Martino, Messina, Italy; Massimo Raffaele Mannarino, Internal Medicine, Angiology and Arteriosclerosis Diseases. Department of Medicine and Surgery. University of Perugia, Perugia, Italy; Bucci Marco, Clinica Medica, Centro di riferimento regionale per le Dislipidemie, Ospedale Policlinico S.S. Annunziata, Chieti, Italy; Lorenzo Maroni, Ambulatorio ipertensione dislipidemie rischio cardiovascolare, ASST Valle Olona, Ospedale di Gallarate, Gallarate, Italy, Ospedale di Busto Arsizio, Busto Arsizio, Italy; Ilenia Minicocci, Dipartimento di Medicina Traslazionale e di Precisione, Sapienza Università di Roma—A. U. O Policlinico Umberto I, Rome, Italy; Giuliana Mombelli, Centro Dislipidemie ASST Grande Ospedale Metropolitano Niguarda, Milan, Italy; Sandro Muntoni, Dipartimento di Scienze Biomediche, Università degli Studi di Cagliari and Centro per le Malattie Dismetaboliche e l’Arteriosclerosi, Associazione ME.DI.CO. Onlus Cagliari, Cagliari, Italy; Fabio Nascimbeni, U.O. Medicina interna metabolica, Centro dislipidemie e malattie metaboliche rare, Ospedale Civile Baggiovara, AOU di Modena, Modena, Italy; Elena Olmastroni, Servizio di Epidemiologia e Farmacologia Preventiva (SEFAP), Dipartimento di Science Farmacologiche e Biomolecolari, Università degli Studi di Milano, Milan, Italy; Gianfranco Parati, IRCCS Istituto Auxologico Italiano and Dipartimento di Medicina e Chirurgia, Università di Milano-Bicocca, Milan, Italy; Angelina Passaro, Department of Translational Medicine, University of Ferrara, Ferrara, Italy and Research and Innovation Section, University Hospital of Ferrara Arcispedale Sant'Anna, Ferrara, Italy; Chiara Pavanello, Centro Dislipidemie ASST Grande Ospedale Metropolitano Niguarda, Milan, Italy and Centro Grossi Paoletti, Dipartimento di Scienze Farmacologiche e Biomolecolari, Università degli Studi di Milano, Milano, Italy; Cristina Pederiva, U.O. Clinica Pediatrica, Servizio Clinico Dislipidemie per lo Studio e la Prevenzione dell’Aterosclerosi in età Pediatrica, ASST-Santi Paolo e Carlo, Milan, Italy; Fabio Pellegatta, Centro per lo Studio dell'Aterosclerosi, IRCCS MultiMedica, Sesto San Giovanni (Milan), Italy and Centro per lo Studio dell’Aterosclerosi, Ospedale E. Bassini, Cinisello Balsamo, Milan, Italy; Francesco Massimo Perla, Dipartimento Materno Infantile e Scienze Urologiche—Sapienza Università, A.O. Policlinico Umberto I, Rome, Italy; Medicina Generale, Ospedale di Trecenta, Trecenta, Rovigo, Italy; Matteo Pirro, Internal Medicine, Angiology and Arteriosclerosis Diseases. Department of Medicine and Surgery. University of Perugia, Perugia, Italy; Livia Pisciotta, IRCCS Ospedale policlinico San Martino UOSD Dietetica e Nutrizione Clinica and Dipartimento di Medicina Interna, Università di Genova, Genova, Italy; Arturo Pujia, A.O.U. Mater Domini, Catanzaro, UOC di Nutrizione Clinica, Ambulatorio Dislipidemie, Catanzaro, Italy; Francesco Purrello, Department of Clinical and Experimental Medicine, University of Catania, Ospedale Garibaldi, Catania, Italy; Elisabetta Rinaldi, U.O. Endocrinologia, Diabetologia e Malattie del Metabolismo, Centro regionale specializzato per la diagnosi e terapia delle dislipidemie e aferesi terapeutica and A.O. Universitaria Integrata di Verona, Verona, Italy; Riccardo Sarzani, Internal Medicine and Geriatrics, Department of Clinical and Molecular Sciences, University “Politecnica delle Marche” and IRCCS-INRCA, Ancona, Italy; Roberto Scicali, Department of Clinical and Experimental Medicine, University of Catania, Ospedale Garibaldi, Catania, Italy; Patrizia Suppressa, U.O. di Medicina Interna e Geriatria “C. Frugoni” and Centro di Assistenza e Ricerca Malattie Rare, A.O. Universitaria Policlinico Consorziale, Università degli Studi di Bari “Aldo Moro”, Bari, Italy; Patrizia Tarugi, Department of Life Sciences, University of Modena and Reggio Emilia, Modena, Italy; Sabrina Verachtert, Department of Clinical and Experimental Medicine—Lipid Center—University Hospital G. Martino, Messina, Italy; Giovanni Battista Vigna, Medicina Generale, Ospedale di Trecenta, Trecenta, Rovigo, Italy; Josè Pablo Werba, U.O. Ambulatorio Prevenzione Aterosclerosi, IRCCS Centro Cardiologico Monzino, Milan, Italy; Alberto Zambon, Dipartimento di Medicina, Università di Padova, Padua, Italy; Sabina Zambon, Dipartimento di Medicina, Università di Padova, Padua, Italy; Maria Grazia Zenti, Servizio di Diabetologia e Malattie Metaboliche, Ospedale P. Pederzoli, Peschiera del Garda, Verona, Italy.

## Data Availability

The original contributions presented in the study are included in the article/Supplementary Material. Further inquiries can be directed to Manuela Casula, manuela.casula@unimi.it.
