# Corrigendum: The role of registers in increasing knowledge and improving management of children and adolescents affected by familial hypercholesterolemia: The LIPIGEN pediatric group

**DOI:** 10.3389/fgene.2023.1113454

**Published:** 2023-04-18

**Authors:** Marta Gazzotti, Manuela Casula, Stefano Bertolini, Maria Elena Capra, Elena Olmastroni, Alberico Luigi Catapano, Cristina Pederiva, LIPIGEN Paediatric Group

**Affiliations:** ^1^ SISA Foundation, Milan, Italy; ^2^ IRCCS MultiMedica, Sesto San Giovanni (Milan), Italy; ^3^ Department of Pharmacological and Biomolecular Sciences, Epidemiology and Preventive Pharmacology Service (SEFAP), University of Milan, Milan, Italy; ^4^ Department of Internal Medicine, University of Genova, Genova, Italy; ^5^ Centre for Paediatric Dyslipidaemias, Paediatrics and Neonatology Unit, Guglielmo da Saliceto Hospital, Piacenza, Italy; ^6^ Clinical Service for Dyslipidaemias, Study and Prevention of Atherosclerosis in Childhood, Paediatrics Unit, ASST-Santi Paolo e Carlo, Milan, Italy

**Keywords:** familial hypercholesterolemia, pediatric cohort, genetic diagnosis, pathology register, clinical diagnosis, cardiovascular genetics

In the published article, there was an error in the author list, and **LIPIGEN Paediatric Group** was erroneously excluded. The corrected author list appears below.

“Marta Gazzotti^1^, Manuela Casula^2,3^, Stefano Bertolini^4^, Maria Elena Capra^5^, Elena Olmastroni^3^, Alberico Luigi Catapano^2,3^, Cristina Pederiva^6^ and the LIPIGEN Paediatric Group

1SISA Foundation, Milan, Italy

2IRCCS MultiMedica, Sesto San Giovanni (Milan), Italy

3Department of Pharmacological and Biomolecular Sciences, Epidemiology and Preventive Pharmacology Service (SEFAP), University of Milan, Milan, Italy

4Department of Internal Medicine, University of Genova, Genova, Italy

5Centre for Paediatric Dyslipidaemias, Paediatrics and Neonatology Unit, Guglielmo da Saliceto Hospital, Piacenza, Italy

6Clinical Service for Dyslipidaemias, Study and Prevention of Atherosclerosis in Childhood, Paediatrics Unit, ASST-Santi Paolo e Carlo, Milan, Italy”

## LIPIGEN Paediatric Group

Massimiliano Allevi, Internal Medicine and Geriatrics, Department of Clinical and Molecular Sciences, University “Politecnica delle Marche” and IRCCS-INRCA, Ancona, Italy; Marcello Arca, Dipartimento di Medicina Traslazionale e di Precisione, Sapienza Università di Roma—A. U. O Policlinico Umberto I, Rome, Italy; Renata Auricchio, Dipartimento di Scienze Mediche Traslazionali, AOU Policlinico Federico II, Naples, Italy; Maurizio Averna, Dipartimento di Promozione della Salute, Materno-Infantile, di Medicina Interna e Specialistica di Eccellenza, Università degli Studi di Palermo, Palermo, Italy; Davide Baldera, Dipartimento di Scienze Biomediche, Università degli Studi di Cagliari and Centro per le Malattie Dismetaboliche e l’Arteriosclerosi, Associazione ME.DI.CO. Onlus Cagliari, Cagliari, Italy; Giuseppe Banderali, U.O. Clinica Pediatrica, Servizio Clinico Dislipidemie per lo Studio e la Prevenzione dell’Aterosclerosi in età Pediatrica, ASST-Santi Paolo e Carlo, Milan, Italy; Andrea Bartuli, UOC Malattie Rare e Genetica Medica, Ospedale Pediatrico Bambino Gesù, IRCCS, Rome, Italy; Stefano Bertolini, Department of Internal Medicine, University of Genova, Genova, Italy; Giacomo Biasucci, Centro Dislipidemie in Età Evolutiva, U.O. Pediatria e Neonatologia, Ospedale Guglielmo da Saliceto, Piacenza, Italy; Claudio Borghi, U.O. di Medicina Interna Cardiovascolare, Centro Aterosclerosi, Ambulatorio Dislipidemie, IRCCS S. Orsola Ospedale Policlinico S. Orsola-Malpighi, Bologna, Italy; Patrizia Bruzzi, U.O.C. Pediatria, Azienda Ospedaliero Universitaria di Modena, Modena, Italy; Raffaele Buganza, Paediatric Endocrinology, Department of Public Health and Paediatric Sciences, Turin University, Turin, Italy; Paola Sabrina Buonuomo, UOC Malattie Rare e Genetica Medica, Ospedale Pediatrico Bambino Gesù, IRCCS, Rome, Italy; Paolo Calabrò, U.O.C. Cardiologia Clinica a Direzione Universitaria e U.T.I.C., A.O.R.N. “Sant'Anna e San Sebastiano”, Caserta, Italy and Dipartimento di Scienze Mediche Traslazionali, Università degli Studi della Campania "Luigi Vanvitelli”, Naples, Italy; Sebastiano Calandra, Department of Biomedical, Metabolic and Neural Sciences, University of Modena and Reggio Emilia, Modena, Italy; Maria Elena Capra, Centro Dislipidemie in Età Evolutiva, U.O. Pediatria e Neonatologia, Ospedale Guglielmo da Saliceto, Piacenza, Italy; Francesca Carubbi, U.O. Medicina interna metabolica, Centro dislipidemie e malattie metaboliche rare, Ospedale Civile Baggiovara, AOU di Modena, Modena, Italy; Manuela Casula, Dipartimento di Scienze Farmacologiche e Biomolecolari, Università degli Studi di Milano, and IRCCS MultiMedica, Sesto San Giovanni (Milan), Italy; Alberico Luigi Catapano, Dipartimento di Scienze Farmacologiche e Biomolecolari, Università degli Studi di Milano, and IRCCS MultiMedica, Sesto San Giovanni (Milan), Italy; Arturo Cesaro, U.O.C. Cardiologia Clinica a Direzione Universitaria e U.T.I.C., A.O.R.N. “Sant'Anna e San Sebastiano”, Caserta, Italy and Dipartimento di Scienze Mediche Traslazionali, Università degli Studi della Campania “Luigi Vanvitelli”, Naples, Italy; Francesco Cipollone, Clinica Medica, Centro di riferimento regionale per le Dislipidemie, Ospedale Policlinico S.S. Annunziata, Chieti, Italy; Nadia Citroni, Centro Dislipidemie e Aterosclerosi, UOC Medicina Interna, Ospedale di Trento, Trento, Italy; Giuseppe Covetti, U.O. Medicina Interna 2, Centro per le malattie da arteriosclerosi, AORN Cardarelli, Naples, Italy; Annalaura Cremonini, IRCCS Ospedale policlinico San Martino UOSD Dietetica e Nutrizione Clinica and Dipartimento di Medicina Interna, Università di Genova, Genova, Italy; Sergio D’Addato, U.O. di Medicina Interna Cardiovascolare, Centro Aterosclerosi, Ambulatorio Dislipidemie, IRCCS S. Orsola Ospedale Policlinico S. Orsola-Malpighi, Bologna, Italy; Maria Del Ben, Dipartimento Scienze Cliniche, Internistiche, Anestesiologiche e Cardiovascolari - Sapienza Università, A.O. Policlinico Umberto I, Rome, Italy; Maria Donata Di Taranto, Dipartimento di Medicina Molecolare e Biotecnologie Mediche, Università degli studi di Napoli Federico II and CEINGE Biotecnologie Avanzate s.c.a.r.l., Naples, Italy; Giuliana Fortunato, Dipartimento di Medicina Molecolare e Biotecnologie Mediche, Università degli studi di Napoli Federico II and CEINGE Biotecnologie Avanzate s.c.a.r.l., Naples, Italy; Roberto Franceschi, UOC Pediatria, Ospedale di Trento, Trento, Italy; Federica Galimberti, IRCCS MultiMedica, Sesto San Giovanni (Milan), Italy; Marta Gazzotti, Fondazione SISA (Società Italiana per lo Studio dell’Aterosclerosi), Milan, Italy; Simonetta Genovesi, IRCCS Istituto Auxologico Italiano and Dipartimento di Medicina e Chirurgia, Università di Milano-Bicocca, Milan, Italy; Antonina Giammanco, Dipartimento di Promozione della Salute, Materno-Infantile, di Medicina Interna e Specialistica di Eccellenza, Università degli Studi di Palermo, Palermo, Italy; Liliana Grigore, Centro per lo Studio dell'Aterosclerosi, IRCCS MultiMedica, Sesto San Giovanni (Milan), Italy and Centro per lo Studio dell’Aterosclerosi, Ospedale E. Bassini, Cinisello Balsamo, Milan, Italy; Ornella Guardamagna, Paediatric Endocrinology, Department of Public Health and Paediatric Sciences, Turin University, Turin, Italy; Arcangelo Iannuzzi, U.O. Medicina Interna 2, Centro per le malattie da arteriosclerosi, AORN Cardarelli, Naples, Italy; Gabriella Iannuzzo, Dipartimento di Medicina Clinica e Chirurgia, Centro Coordinamento regionale per le Iperlipidemie, AOU Policlinico Federico II, Naples, Italy; Lidia Lascala, AOU Mater Domini, Catanzaro; Fabiana Locatelli, Ambulatorio ipertensione dislipidemie rischio cardiovascolare, ASST Valle Olona, Ospedale di Gallarate, Gallarate, Italy, Ospedale di Busto Arsizio, Busto Arsizio, Italy; Lorenzo Iughetti, U.O.C. Pediatria, Azienda Ospedaliero Universitaria di Modena, Modena, Italy; Sara Madaghiele, U.O. di Medicina Interna e Geriatria “C. Frugoni” and Centro di Assistenza e Ricerca Malattie Rare, A.O. Universitaria Policlinico Consorziale, Università degli Studi di Bari “Aldo Moro”, Bari, Italy; Giuseppe Mandraffino, Department of Clinical and Experimental Medicine—Lipid Center—University Hospital G. Martino, Messina, Italy; Massimo Raffaele Mannarino, Internal Medicine, Angiology and Arteriosclerosis Diseases. Department of Medicine and Surgery. University of Perugia, Perugia, Italy; Bucci Marco, Clinica Medica, Centro di riferimento regionale per le Dislipidemie, Ospedale Policlinico S.S. Annunziata, Chieti, Italy; Lorenzo Maroni, Ambulatorio ipertensione dislipidemie rischio cardiovascolare, ASST Valle Olona, Ospedale di Gallarate, Gallarate, Italy, Ospedale di Busto Arsizio, Busto Arsizio, Italy; Ilenia Minicocci, Dipartimento di Medicina Traslazionale e di Precisione, Sapienza Università di Roma—A. U. O Policlinico Umberto I, Rome, Italy; Giuliana Mombelli, Centro Dislipidemie ASST Grande Ospedale Metropolitano Niguarda, Milan, Italy; Sandro Muntoni, Dipartimento di Scienze Biomediche, Università degli Studi di Cagliari and Centro per le Malattie Dismetaboliche e l’Arteriosclerosi, Associazione ME.DI.CO. Onlus Cagliari, Cagliari, Italy; Fabio Nascimbeni, U.O. Medicina interna metabolica, Centro dislipidemie e malattie metaboliche rare, Ospedale Civile Baggiovara, AOU di Modena, Modena, Italy; Elena Olmastroni, Servizio di Epidemiologia e Farmacologia Preventiva (SEFAP), Dipartimento di Science Farmacologiche e Biomolecolari, Università degli Studi di Milano, Milan, Italy; Gianfranco Parati, IRCCS Istituto Auxologico Italiano and Dipartimento di Medicina e Chirurgia, Università di Milano-Bicocca, Milan, Italy; Angelina Passaro, Department of Translational Medicine, University of Ferrara, Ferrara, Italy and Research and Innovation Section, University Hospital of Ferrara Arcispedale Sant'Anna, Ferrara, Italy; Chiara Pavanello, Centro Dislipidemie ASST Grande Ospedale Metropolitano Niguarda, Milan, Italy and Centro Grossi Paoletti, Dipartimento di Scienze Farmacologiche e Biomolecolari, Università degli Studi di Milano, Milano, Italy; Cristina Pederiva, U.O. Clinica Pediatrica, Servizio Clinico Dislipidemie per lo Studio e la Prevenzione dell’Aterosclerosi in età Pediatrica, ASST-Santi Paolo e Carlo, Milan, Italy; Fabio Pellegatta, Centro per lo Studio dell'Aterosclerosi, IRCCS MultiMedica, Sesto San Giovanni (Milan), Italy and Centro per lo Studio dell’Aterosclerosi, Ospedale E. Bassini, Cinisello Balsamo, Milan, Italy; Francesco Massimo Perla, Dipartimento Materno Infantile e Scienze Urologiche—Sapienza Università, A.O. Policlinico Umberto I, Rome, Italy; Medicina Generale, Ospedale di Trecenta, Trecenta, Rovigo, Italy; Matteo Pirro, Internal Medicine, Angiology and Arteriosclerosis Diseases. Department of Medicine and Surgery. University of Perugia, Perugia, Italy; Livia Pisciotta, IRCCS Ospedale policlinico San Martino UOSD Dietetica e Nutrizione Clinica and Dipartimento di Medicina Interna, Università di Genova, Genova, Italy; Arturo Pujia, A.O.U. Mater Domini, Catanzaro, UOC di Nutrizione Clinica, Ambulatorio Dislipidemie, Catanzaro, Italy; Francesco Purrello, Department of Clinical and Experimental Medicine, University of Catania, Ospedale Garibaldi, Catania, Italy; Elisabetta Rinaldi, U.O. Endocrinologia, Diabetologia e Malattie del Metabolismo, Centro regionale specializzato per la diagnosi e terapia delle dislipidemie e aferesi terapeutica and A.O. Universitaria Integrata di Verona, Verona, Italy; Riccardo Sarzani, Internal Medicine and Geriatrics, Department of Clinical and Molecular Sciences, University “Politecnica delle Marche” and IRCCS-INRCA, Ancona, Italy; Roberto Scicali, Department of Clinical and Experimental Medicine, University of Catania, Ospedale Garibaldi, Catania, Italy; Patrizia Suppressa, U.O. di Medicina Interna e Geriatria “C. Frugoni” and Centro di Assistenza e Ricerca Malattie Rare, A.O. Universitaria Policlinico Consorziale, Università degli Studi di Bari “Aldo Moro”, Bari, Italy; Patrizia Tarugi, Department of Life Sciences, University of Modena and Reggio Emilia, Modena, Italy; Sabrina Verachtert, Department of Clinical and Experimental Medicine—Lipid Center—University Hospital G. Martino, Messina, Italy; Giovanni Battista Vigna, Medicina Generale, Ospedale di Trecenta, Trecenta, Rovigo, Italy; Josè Pablo Werba, U.O. Ambulatorio Prevenzione Aterosclerosi, IRCCS Centro Cardiologico Monzino, Milan, Italy; Alberto Zambon, Dipartimento di Medicina, Università di Padova, Padua, Italy; Sabina Zambon, Dipartimento di Medicina, Università di Padova, Padua, Italy; Maria Grazia Zenti, Servizio di Diabetologia e Malattie Metaboliche, Ospedale P. Pederzoli, Peschiera del Garda, Verona, Italy.

The authors apologize for this error and state that this does not change the scientific conclusions of the article in any way. The original article has been updated.

