# Supplementary material for: The Role of Registers in Increasing Knowledge and Improving Management of Children and Adolescents Affected by Familial Hypercholesterolemia: the LIPIGEN Pediatric Group
Source: Front Genet. 2022 Jun 20;13:912510. doi: 10.3389/fgene.2022.912510 (PMC9251337; doi:10.3389/fgene.2022.912510)
Supplement: Supplementary file 1 [file DataSheet1.docx]

The role of registers in increasing knowledge and improving management of children and adolescents affected by familial hypercholesterolemia: the LIPIGEN Paediatric Group

Marta Gazzotti^1^, Manuela Casula^2,3^, Stefano Bertolini^4^, Maria Elena Capra^5^, Elena Olmastroni^3^, Alberico Luigi Catapano^2,3^, Cristina Pederiva^6^, on behalf of the LIPIGEN Paediatric Group^‡^

^1^ SISA Foundation, Milan, Italy

^2^ IRCCS MultiMedica, Sesto S. Giovanni (MI), Italy

^3^ Epidemiology and Preventive Pharmacology Service (SEFAP), Department of Pharmacological and Biomolecular Sciences, University of Milan, Milan, Italy

^4^ Department of Internal Medicine, University of Genova, Genova, Italy

^5^ Centre for Paediatric Dyslipidaemias, Paediatrics and Neonatology Unit, Guglielmo da Saliceto Hospital, Piacenza, Italy

^6^ Clinical Service for Dyslipidaemias, Study and Prevention of Atherosclerosis in Childhood, Paediatrics Unit, ASST-Santi Paolo e Carlo, Milan, Italy

*** Correspondence:**Manuela Casula, PhD
Epidemiology and Preventive Pharmacology Service (SEFAP), Department of Pharmacological and Biomolecular Sciences, University of Milan, Via G. Balzaretti, 9, 20133, Milan, Italy
IRCCS MultiMedica, Sesto S. Giovanni (MI), Italy
E-mail: [manuela.casula@unimi.it](mailto:manuela.casula@unimi.it)

# Supplementary Material

**Supplementary Table 1** – Homozygotes for *LDLR* gene variants

| ***LDLR* gene variant** | **Protein change** | **N of subjects** |
| --- | --- | --- |
| c.1646G>A | p.Gly549Asp | 3 |
| c.68-?_1845+?del Exons 2_12del | p.Val23Glyfs*29 | 2 |
| c.1567G>A | p.Val523Met | 2 |
| c.2054C>T | p.Pro685Leu | 2 |
| c.373C>T | p.Gln125* | 1 |
| c.671A>G | p.Asp224Gly | 1 |
| c.940_940+14 | p.Ser306Aspfs*17 | 1 |
| c.1056C>G | p.Cys352Trp | 1 |
| c.1109A>C | p.Asn370Thr | 1 |
| IVS10 c.1586+1G>A | p.[Thr454_Gly529del, p.Gly529_Phe530ins22] | 1 |
| IVS15 c.2311+1G>A | p.[Gln770_Ala771ins30, Lys730fs*17,Thr713_Ala771del] | 1 |
| IVS16 c.2390-1G>A | p.Val797Alafs*155 | 1 |

**Supplementary Table 2** – Compound Heterozygotes carriers of two *LDLR* gene variants

| ***LDLR* gene 1st variant** | **Protein** | ***LDLR* gene 2nd variant** | **Protein** | **N of subjects** |
| --- | --- | --- | --- | --- |
| c.1646G>A | p.Gly549Asp | c.81C>G | p.Cys27Trp | 3 |
| c.1646G>A | p.Gly549Asp | c.1846-?_2311+?del | p.Asp616Leufs*17 | 2 |
| c.1775G>A | p.Gly592Glu | c.1135T>C | p.Cys379Arg | 2 |
| c.1775G>A | p.Gly592Glu | c.265T>C | p.Cys89Arg | 2 |
| c.268G>A | p.Asp90Asn | c.666C>A | p.Cys222* | 1 |
| c.352G>T | p.Asp118Tyr | c.418G>T | p.Glu140* | 1 |
| c.1068T>A+ c.1069_1086dup | p.Asp356Glu/Glu357_Asp362dup | c.1846-1894_ 2140+1498del | p.Asp616Argfs*16 | 1 |
| c.1118G>A | p.Gly373Asp | c.126C>G, | p.Tyr42* | 1 |
| c.1135T>C | p.Cys379Arg | c.1567G>A | p.Val523Met | 1 |
| c.1230G>C | p.Arg410Ser | c.1478_1479delCT | p.Ser493Cisfs*42 | 1 |
| c.1472C>A | p.Thr491Asn | c.535G>A | p.Glu179Lys | 1 |
| c.1775G>A | p.Gly592Glu | c.407A>T | p.Asp136Val | 1 |
| c.1775G>A | p.Gly592Glu | c.694+4_5insT | p.Ala232Glyfs*28 | 1 |

**Supplementary Table 3** – Double Heterozygotes for *LDLR* and *APOB* (N=2) or *LDLR* and *PCSK9* (N=1) gene variants

| **Gene 1st** | ***LDLR* gene**  **variant** | **Protein** | **Gene  2nd** | ***APOB*/*PCSK9***  **gene variant** | **Protein** |
| --- | --- | --- | --- | --- | --- |
| LDLR | c.662A>G | p.Asp221Gly | APOB | c.10580G>A | p.Arg3527Gln |
| LDLR | c.1257C>G | p.Tyr419* | APOB | c.10672C>T | p.Arg3558Cys |
| LDLR | c.1694G>T | p.Gly565Val | PCSK9 | c.-331C>A* | GOF |

*Blesa S et al. J Clin Endocrinol Metab 2008; 93: 3577-3583 (Blesa et al., 2008)

# Reference

Blesa, S., Vernia, S., Garcia-Garcia, A.B., Martinez-Hervas, S., Ivorra, C., Gonzalez-Albert, V., et al. (2008). A new PCSK9 gene promoter variant affects gene expression and causes autosomal dominant hypercholesterolemia. *J Clin Endocrinol Metab* 93(9)**,** 3577-3583. doi: 10.1210/jc.2008-0269.

**Supplementary Figure 1** – Distribution of untreated LDL-C levels by age among the carriers of the same causative variant. The panel (A) shows data related to carriers of c.1646G>A p.Gly549Asp, the panel (B) of c.1775G>A p.Gly592Glu, and the panel (C) of c.662A>G p.Asp221Gly.

**(A) Untreated LDL-C levels in carriers of c.1646G>A p.Gly549Asp**

**(B) Untreated LDL-C levels in carriers of c.1775G>A p.Gly592Glu**

**(C) Untreated LDL-C levels in carriers of c.662A>G p.Asp221Gly**

**Appendix. LIPIGEN Paediatric group**

**MEMBERS OF THE LIPIGEN STEERING COMMETTEE**: Arca Marcello^1^, Averna Maurizio^2^, Bertolini Stefano^3^, Calandra Sebastiano^4^, Catapano Alberico Luigi^5^, Tarugi Patrizia^6^. **PRINCIPAL INVESTIGATORS**: **Coordinators**: Pederiva Cristina^26^, Capra Maria Elena^8^. **Participant Centers**: Arca Marcello^1^, Averna Maurizio^2^, Bartuli Andrea^7^, Biasucci Giacomo^8^, Borghi Claudio^9^, Calabrò Paolo^10^, Carubbi Francesca^11^, Cipollone Francesco^12^, Citroni Nadia^13^, Del Ben Maria^14^, Fortunato Giuliana^15^, Guardamagna Ornella^16^, Iannuzzi Arcangelo^17^, Iannuzzo Gabriella^18^, Iughetti Lorenzo^19^, Mandraffino Giuseppe^20^, Maroni Lorenzo^21^, Mombelli Giuliana^22^, Muntoni Sandro^23^, Parati Gianfranco^24^, Passaro Angelina^25^, Pederiva Cristina^26^, Pellegatta Fabio^27^, Pirro Matteo^28^, Pisciotta Livia^29^, Pujia Arturo^30^, Purrello Francesco^31^, Sarzani Riccardo^32^, Suppressa Patrizia^33^, Werba Josè Pablo^34^, Zambon Sabina^35^, Zenti Maria Grazia^36^. **Participant COLLABORATORS**: Allevi Massimiliano^32^, Auricchio Renata^37^, Baldera Davide^23^, Banderali Giuseppe^26^, Bruzzi Patrizia^19^, Bucci Marco^12^, Buganza Raffaele^16^, Buonuomo Paola Sabrina^7^, Cesaro Arturo^10^, Covetti Giuseppe^17^, Cremonini Annalaura^29^, D’Addato Sergio^9^, Di Taranto Maria Donata^15^, Franceschi Roberto^38^, Genovesi Simonetta^24^, Giammanco Antonina^2^, Grigore Liliana^27^, Lascala Lidia^45^, Locatelli Fabiana^21^, Madaghiele Sara^33^, Mannarino Massimo Raffaele^28^, Minicocci Ilenia^1^, Nascimbeni Fabio^11^, Pavanello Chiara^39^, Perla Francesco Massimo^40^, Rinaldi Elisabetta^46^, Scicali Roberto^31^, Verachtert Sabrina^20^, Vigna Giovanni Battista^41^, Zambon Alberto^35^.

**STUDY COORDINATING GROUP:** Catapano Alberico Luigi^5^, Casula Manuela^5^, Galimberti Federica^42^, Gazzotti Marta^43^, Olmastroni Elena^44^.

**Affiliations**: ^1^Dipartimento di Medicina Traslazionale e di Precisione, Sapienza Università di Roma -  A. U. O Policlinico Umberto I, Rome, Italy; ^2^Dipartimento di Promozione della Salute, Materno-Infantile, di Medicina Interna e Specialistica di Eccellenza, Università degli Studi di Palermo, Palermo, Italy; ^3^Department of Internal Medicine, University of Genova, Genova, Italy; ^4^Department of Biomedical, Metabolic and Neural Sciences, University of Modena and Reggio Emilia, Modena, Italy; ^5^Dipartimento di Scienze Farmacologiche e Biomolecolari, Università degli Studi di Milano, and IRCCS Multimedica, Milan, Italy; ^6^Department of Life Sciences, University of Modena and Reggio Emilia, Modena, Italy; ^7^UOC Malattie Rare e Genetica Medica, Ospedale Pediatrico Bambino Gesù, IRCCS, Rome, Italy; ^8^Centro Dislipidemie in Età Evolutiva, U.O. Pediatria e Neonatologia, Ospedale Guglielmo da Saliceto, Piacenza, Italy; ^9^U.O. di Medicina Interna Cardiovascolare, Centro Aterosclerosi, Ambulatorio Dislipidemie, IRCCS S. Orsola Ospedale Policlinico S. Orsola-Malpighi, Bologna, Italy; ^10^U.O.C. Cardiologia Clinica a Direzione Universitaria e U.T.I.C., A.O.R.N. "Sant'Anna e San Sebastiano", Caserta, Italy and Dipartimento di Scienze Mediche Traslazionali, Università degli Studi della Campania "Luigi Vanvitelli", Naples, Italy; ^11^U.O. Medicina interna metabolica, Centro dislipidemie e malattie metaboliche rare, Ospedale Civile Baggiovara, AOU di Modena, Modena, Italy; ^12^Clinica Medica, Centro di riferimento regionale per le Dislipidemie, Ospedale Policlinico S.S. Annunziata, Chieti, Italy; ^13^Centro Dislipidemie e Aterosclerosi, UOC Medicina Interna, Ospedale di Trento, Trento, Italy; ^14^Dipartimento Scienze Cliniche, Internistiche, Anestesiologiche e Cardiovascolari - Sapienza Università, A.O. Policlinico Umberto I, Rome, Italy; ^15^Dipartimento di Medicina Molecolare e Biotecnologie Mediche, Università degli studi di Napoli Federico II and CEINGE Biotecnologie Avanzate s.c.a.r.l., Naples, Italy; ^16^Paediatric Endocrinology, Department of Public Health and Paediatric Sciences, Turin University, Turin, Italy; ^17^U.O. Medicina Interna 2, Centro per le malattie da arteriosclerosi, AORN Cardarelli, Naples, Italy; ^18^Dipartimento di Medicina Clinica e Chirurgia, Centro Coordinamento regionale per le Iperlipidemie, AOU Policlinico Federico II, Naples, Italy; ^19^U.O.C. Pediatria, Azienda Ospedaliero Universitaria di Modena, Modena, Italy; ^20^Department of Clinical and Experimental Medicine - Lipid Center - University Hospital G. Martino, Messina, Italy; ^21^Ambulatorio ipertensione dislipidemie rischio cardiovascolare, ASST Valle Olona, Ospedale di Gallarate, Gallarate, Italy, Ospedale di Busto Arsizio, Busto Arsizio, Italy; ^22^Centro Dislipidemie ASST Grande Ospedale Metropolitano Niguarda, Milan, Italy; ^23^Dipartimento di Scienze Biomediche, Università degli Studi di Cagliari and Centro per le Malattie Dismetaboliche e l’Arteriosclerosi, Associazione ME.DI.CO. Onlus Cagliari, Cagliari, Italy; ^24^IRCCS Istituto Auxologico Italiano and Dipartimento di Medicina e Chirurgia, Università di Milano-Bicocca, Milan, Italy; ^25^Department of Translational Medicine, University of Ferrara, Ferrara, Italy and Research and Innovation Section, University Hospital of Ferrara Arcispedale Sant'Anna, Ferrara, Italy; ^26^U.O. Clinica Pediatrica, Servizio Clinico Dislipidemie per lo Studio e la Prevenzione dell’Aterosclerosi in età Pediatrica, ASST-Santi Paolo e Carlo, Milan, Italy; ^27^Centro per lo Studio dell'Aterosclerosi, IRCCS Multimedica, Sesto San Giovanni, Italy and Centro per lo Studio dell’Aterosclerosi, Ospedale E. Bassini, Cinisello Balsamo, Milan, Italy; ^28^Internal Medicine, Angiology and Arteriosclerosis Diseases. Department of Medicine and Surgery. University of Perugia, Perugia, Italy; ^29^IRCCS Ospedale policlinico San Martino UOSD Dietetica e Nutrizione Clinica and Dipartimento di Medicina Interna, Università di Genova, Genova, Italy; ^30^A.O.U. Mater Domini, Catanzaro, UOC di Nutrizione Clinica, Ambulatorio Dislipidemie, Catanzaro, Italy; ^31^Department of Clinical and Experimental Medicine, University of Catania, Ospedale Garibaldi, Catania, Italy; ^32^Internal Medicine and Geriatrics, Department of Clinical and Molecular Sciences, University “Politecnica delle Marche” and IRCCS-INRCA, Ancona, Italy; ^33^U.O. di Medicina Interna e Geriatria “C. Frugoni” and Centro di Assistenza e Ricerca Malattie Rare, A.O. Universitaria Policlinico Consorziale, Università degli Studi di Bari "Aldo Moro", Bari, Italy; ^34^U.O. Ambulatorio Prevenzione Aterosclerosi, IRCCS Centro Cardiologico Monzino, Milan, Italy; ^35^Dipartimento di Medicina, Università di Padova, Padua, Italy; ^36^Servizio di Diabetologia e Malattie Metaboliche, Ospedale P. Pederzoli, Peschiera del Garda, Verona, Italy; ^37^Dipartimento di Scienze Mediche Traslazionali, AOU Policlinico Federico II, Naples, Italy; ^38^UOC Pediatria, Ospedale di Trento, Trento, Italy; ^39^Centro Dislipidemie ASST Grande Ospedale Metropolitano Niguarda, Milan, Italy and Centro Grossi Paoletti, Dipartimento di Scienze Farmacologiche e Biomolecolari, Università degli Studi di Milano, Milano, Italy; ^40^Dipartimento Materno Infantile e Scienze Urologiche - Sapienza Università, A.O. Policlinico Umberto I, Rome, Italy; ^41^Medicina Generale, Ospedale di Trecenta, Trecenta, Rovigo, Italy; ^42^IRCCS Multimedica, Sesto San Giovanni (MI), Milano, Italy; ^43^Fondazione SISA (Società Italiana per lo Studio dell’Aterosclerosi), Milan, Italy; ^44^Servizio Universitario di Epidemiologia e Farmacologia Preventiva (SEFAP), Dipartimento di Science Farmacologiche e Biomolecolari, Università degli Studi di Milano, Milan, Italy; ^45^AOU Mater Domini, Catanzaro; ^46^U.O. Endocrinologia, Diabetologia e Malattie del Metabolismo, Centro regionale specializzato per la diagnosi e terapia delle dislipidemie e aferesi terapeutica and A.O. Universitaria Integrata di Verona, Verona, Italy
